# Neighbourhood fast food exposure and consumption: the mediating role of neighbourhood social norms

**DOI:** 10.1186/s12966-020-00969-w

**Published:** 2020-05-13

**Authors:** Sofie van Rongen, Maartje P. Poelman, Lukar Thornton, Gavin Abbott, Meng Lu, Carlijn B. M. Kamphuis, Kirsten Verkooijen, Emely de Vet

**Affiliations:** 1 Consumption and Healthy Lifestyles Group, Wageningen University & Research, Hollandseweg 1, 6706 KN Wageningen, the Netherlands; 2 Department of Human Geography, Utrecht University, Princetonlaan 8a, 3584 CB Utrecht, The Netherlands; 3 Institute for Physical Activity and Nutrition, School of Exercise and Nutrition Sciences, Deakin University, 221 Burwood Highway, Burwood, VIC 3125 Australia; 4 Department of Physical Geography, Utrecht University, Princetonlaan 8a, 3584 CB Utrecht, The Netherlands; 5 Department of Interdisciplinary Social Science, Utrecht University, Padualaan 14, 3584 CH Utrecht, the Netherlands; 6 Health and Society Group, Wageningen University & Research, Hollandseweg 1, 6706 KN Wageningen, the Netherlands

**Keywords:** Neighbourhood, Food environment, Fast food outlets, Fast food exposure, Fast food consumption, Social norms, Mediation analysis

## Abstract

**Background:**

The association between the residential fast food environment and diet has gained growing attention. However, why the food environment affects food consumption is under-examined. This study aimed to investigate neighbourhood social norms with respect to fast food consumption as a potential mediating pathway between residential fast food outlet exposure and residents’ fast food consumption.

**Methods:**

A correlational study was conducted in which a nationwide sample of 1038 respondents living across The Netherlands completed a survey. Respondents reported their fast food consumption (amount/week) as well as perceived descriptive and injunctive norms regarding fast food consumption in their neighbourhood. Fast food outlet exposure was measured by the average count of fast food outlets within a 400 m walking distance buffer around the zip-codes of the respondents, using a retail outlet database. Regression models were used to assess associations between residential fast food outlet exposure, fast food consumption, and social norm perceptions, and a bootstrapping procedure was used to test the indirect -mediation- effect. Separate analyses were performed for descriptive norms and injunctive norms.

**Results:**

There was no overall or direct association between residential fast food outlet exposure and residents’ fast food consumption. However, fast food outlet exposure was positively associated with neighbourhood social norms (descriptive and injunctive) regarding fast food consumption, which in turn were positively associated with the odds of consuming fast food. Moreover, results of the bootstrapped analysis provided evidence of indirect effects of fast food outlet exposure on fast food consumption, via descriptive norms and injunctive norms.

**Conclusions:**

In neighbourhoods with more fast food outlets, residents were more likely to perceive fast food consumption in the neighbourhood as more common and appropriate. In turn, stronger neighbourhood social norms were associated with higher fast food consumption. Acknowledging the correlational design, this study is the first that implies that neighbourhood social norms may be a mediating pathway in the relation between the residential fast food environment and fast food consumption. Future research may examine the role of neighbourhood social norms in other contexts and explore how the changing food environment may shift our consumption norms.

## Background

Following the rapid increase in the number of fast food outlets in the past decades [1–3], the relationship between the fast food environment and diet and health outcomes has gained societal attention (e.g. [4–6]). Evidence of an association between neighbourhood exposure to fast food outlets and both diet and health outcomes is however mixed [7–9]. Despite increasing calls and plans to ban fast food outlets in certain areas in a bid to curb obesity, it remains poorly understood *how* the food environment relates to food consumption and there has been a call for research to examine pathways that may explain potential relationships [9–15]. Although various mediating factors have been proposed (e.g. taste preferences, food preparation skills, perceptions of the food environment [10, 14]), studies investigating specific pathways are scarce.

It is well established that the social environment exerts a powerful influence on people’s perceptions and behaviours [16]. People are influenced by others’ behaviours and values to establish what is a correct (informational or descriptive social norm influence) or appropriate (normative or injunctive social norm influence) behaviour [17, 18]. There is a growing body of evidence of social norm influences on dietary behaviour (see for reviews [19–21]), including fast food consumption. More specifically, a cross-sectional study showed that descriptive social norm perceptions regarding others eating fast food was associated with fast food consumption [22]. Yet, the social aspects of the neighbourhood food environment (e.g. eating appropriateness standards, situational norms including social facilitation and modelling of food intake) are understudied [23–25]. Moreover, scholars have treated the physical and social food environment as if these are two independent environmental influences on food consumption [26–28]. However, there are indications that these influences are linked; specifically, that the physical food environment may contribute to social norms regarding appropriate eating [23, 29], thereby affecting food choices [19, 20, 30, 31]. For example, building on social practice theory [32, 33], a qualitative study of fast food neighbourhood perceptions revealed that fast food outlets became normalised for those living near them [29]. Yet, it has not quantitatively been studied whether the neighbourhood-level food environment is associated with social norms, that may in turn be associated with food consumption. We refer to these social norms as ‘neighbourhood social norms’, i.e. perceptions about what other people in the neighbourhood consume and what is normal or appropriate consumption in the neighbourhood.

Various visual aspects within the neighbourhood may form neighbourhood norms about appropriate fast food consumption. For example, people are exposed to fast food outlets, delivery vehicles, individuals purchasing and/or eating fast food at these outlets or on-street, and traces/rubbish of fast food consumption. Hence, both others’ fast food consumption-related behaviours and physical aspects of the neighbourhood may form input for residents’ fast food norm perceptions. Yet, it remains unknown if these elements contribute to fast food norm perceptions, and if so, whether these norm perceptions influence fast food consumption. The present study aimed to investigate to what extent perceived neighborhood social norms towards fast food mediated the association between exposure to fast food outlets in the residential environment and fast food consumption among a nationwide adult sample in the Netherlands. We hypothesized that a higher residential exposure to fast food outlets is associated with more positive neighbourhood social norms regarding common and appropriate fast food consumption (i.e. descriptive and injunctive norms, respectively). In turn, it was hypothesized that the relation between fast food outlet exposure and fast food consumption is mediated through these neighbourhood social norms.

## Methods

### Participants, design and procedure

A nationwide sample living across the Netherlands was recruited by a panel bureau (Flycatcher [34]). The aim was to reach a sample size of 1000 respondents, based on the maximum budget available. Taken into account an expected response rate of 50%, an initial sample of 1988 respondents were emailed an invitation to participate in the survey. Respondents were given 7 days to complete each survey. A reminder email was sent to non-responders one day before the call closed. Inclusion criteria were age 25–60 years and not currently enrolled in education. Eligible respondents from the panel were selected on household income to have an equal proportion of low- middle- and high-incomes. A total number of 387, 338, and 330 respondents in these respective income groups completed our survey, which resulted in a total sample of 1055 (response rate = 53%). In comparison with records from 2018 from Statistics Netherlands [35], this sample was representative for the Dutch population aged 25–60 years with respect to sex, age, education level and province. Seventeen respondents were excluded because they provided a non-existing postcode or because fast food outlet data or area-level income data was missing, resulting in an analytic sample of 1038 respondents (mean age = 45.5, SD = 10.3, 58% female, 95% Dutch ethnicity). Twenty-two (2.1%) respondents had the same postcode. This study has a correlational survey design, where the first survey assessed demographics and neighbourhood norms, and a second survey four weeks later assessed fast food consumption (response rate = 59 and 79%, respectively). Data were collected in January and February 2019. Ethical approval was granted by the ethics committee of the faculty of Bèta-Geo Sciences of Utrecht University, the Netherlands (GEO FETC18–014).

### Fast food outlet data

Addresses of fast food outlets were obtained from the Locatus database (2017), which contains independently and objectively recorded retail information of all outlets in the Netherlands through annual on-site surveys. Data were extracted from three retail categories typically selling fast food: 1) fast-food outlets (e.g. McDonald’s, local “snackbar”), 2) delivery/take-away outlets (e.g. Chinese, pizza); 3) grillroom/kebab outlets. These three retail categories included chain and non-chain outlets selling quickly prepared and served, mainly energy-dense foods for in-store consumption and/or takeaway and/or delivery.

### Measures

#### Outcome measure: fast food consumption

Frequency consumption of fast foods was estimated by two questions asking how frequently during the last four weeks respondents (1) consumed fast foods within a fast food restaurant or through take away (i.e., not delivery) and (2) had fast food delivered from a fast food restaurant. Examples of fast food outlets were given (“Mc Donalds/Burger King/KFC, Febo, snack bar, grillroom (kebab, Turkish pizza, shawarma), New York Pizza, and other fast food outlets (pizza, Chinese, tacos)”. The delivery item also mentioned examples of delivery services (“Takeaway, Ubereats, Foodora, Deliveroo, or the delivery service of the restaurant itself”). There were nine response categories: ‘never or less than once a month’, ‘1-3 times a month’, ‘one day/week’, ‘2 days /week’, ‘3 days /week’, ‘4 days /week’, ‘5 days /week’, 6 ‘days /week’, ‘7 days /week (every day)’. Answers for both items were recoded into weekly equivalent measures of 0 days/week and .5 days/week, 1 day/week, etc. These weekly equivalent scores were summed to generate a weekly equivalent total fast food consumption frequency score. Because 73% of the respondents consumed fast foods 1–3 times a month or less, the weekly scores were subsequently converted into three ordinal categories of ‘never or less than once/month’, ‘1–3 times/month’, and ‘at least once per week’.

#### Exposure measure: residential fast food outlet exposure

**Fig. 1:**
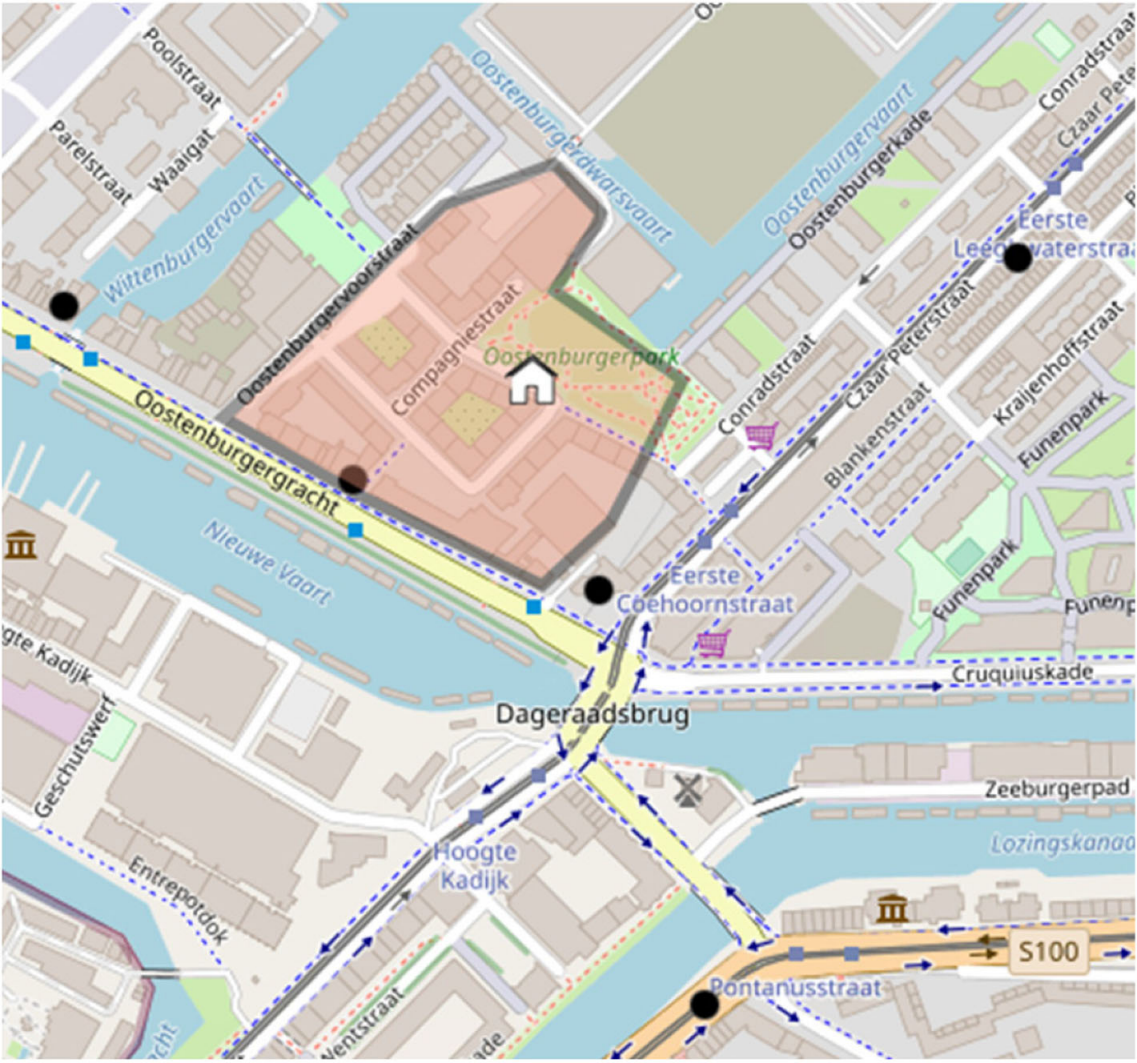
A 400 m walking distance buffer around an address. The black dots represent fast food outlets. Created in Openstreetmap [36]

The cohort was enriched with residential fast food outlet exposure by aggregating all the fast food outlets within a 400 m walking distance buffer from each address in the Netherlands. Figure 1 illustrates how the residential fast food outlet exposure is calculated. The walking distance was calculated based on the Top10L street network [37] with highways removed. The preprocessing (rasterize, resample, mosaic) of the street network was done in ArcGIS (Esri, Redlands, CA, US) and buffer calculations were done in Python [38] and PCRaster [39] environments. For privacy reasons we could not ask respondents to self-report their exact home address in the survey and so we asked participants to report their postcode. This postcode, a combination of 4 digits and 2 letters, contains on average 25 houses and represents the scale of (part of) a street [40, 41]. The average count of fast food outlets within 400 m distance buffers per postcode was calculated and rounded. A continuous measure of the count data was used, which ranged from 0 to 29 fast food outlets.

#### Mediator: perceived neighbourhood social norms

Because of the conceptual distinction between descriptive and injunctive social norms (e.g. [18, 42]), these concepts were measured separately. Descriptive social norms were defined as what the respondent perceived other people in their neighbourhood do in relation to eating fast food, which includes in-store consumption, street consumption, takeaway, and delivery. Descriptive social norms were assessed with the following statements: “I often see other people in my neighbourhood eating or taking away fast food”, and “In my neighbourhood people eat fast food frequently”. Responses were reported on a five-point Likert scale ranging from “strongly disagree” to “strongly agree” (midpoint “neutral (neither disagree or agree)”). A mean score was calculated (Cronbach’s alpha = .89). Injunctive social norms were defined as the respondent’s beliefs regarding approval/appropriateness of eating fast food in their neighbourhood. These were assessed with the statements: “In my neighbourhood it is normal to eat fast food”, “In my neighbourhood it is acceptable to eat fast food”, and “In my neighbourhood it is appropriate to eat fast food”. Response options were the same as those used for the descriptive norms measure. A mean score of these three items was calculated (Cronbach’s alpha = .85). To clarify the (English) term ‘fast food’ to respondents, a definition of fast food was given in Dutch (“Fast food is an unhealthy quick bite”) as well as examples of outlets (identical examples as given with the fast food consumption items). It was also stated that eating fast food entails eating in-store and on-street, as well as takeaway and delivery.

#### Confounders

We used Directed Acyclic Graphs (DAGs, see Additional file 1) to visually represent the assumed causal relationships among the exposure, the outcome, the mediating variables and the covariates [43–46]. This enabled us to carefully select confounders, which are only those factors that may independently affect both the exposure (i.e. fast food outlet exposure) *and* the outcome (i.e. fast food consumption) or an ancestor of these (i.e. neighbourhood norms). This process led us to identify age and area-level income as confounders. Individual level socio-demographic (i.e. ethnicity, household composition) and socio-economic factors (i.e. income level, education level, employment) were assumed to influence fast food outlet exposure only through area-level income, as it is likely that choice of fast food outlet location is more heavily influenced by the collective characteristics of an area, rather than by an individual’s characteristics [47, 48]. Sex was assumed to affect fast food consumption but not exposure or neighbourhood norms. Area-level income was obtained from Statistics Netherlands [49] and was measured as postcode-4 level household equivalent income in 2015, on a continuous scale. A postcode-4 level contains on average 2216 addresses, although there is large variation [50].

### Statistical analyses

**Fig. 2:**
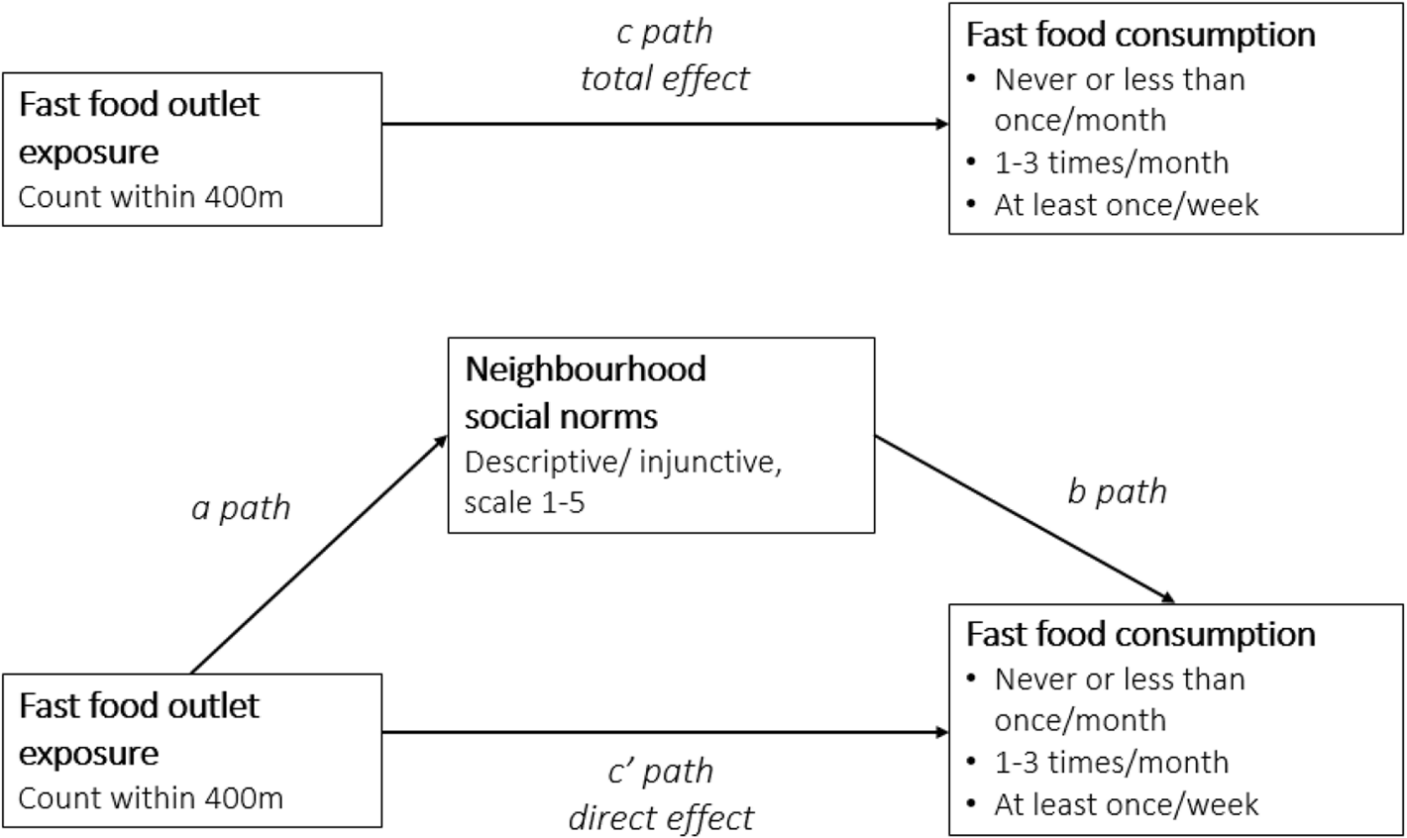
Overview of the mediation model including all pathways. Separate mediation analyses were performed for the two potential mediators (i.e., descriptive norms and injunctive norms). All analyses were controlled for age and area-level income

To test potential indirect effects of fast food outlet exposure on fast food consumption via neighbourhood social norm perceptions, mediation analyses were performed using Stata 13 IC [51]. Separate mediation analyses were conducted for the two potential mediators (i.e., descriptive norms and injunctive norms). The hypothesised mediation model is shown in Fig. 2. First, an ordinal logistic regression model was used to test the total effect of fast food outlet exposure on the outcome variable fast food consumption (c path). Second, a linear regression model was fitted to test the association between fast food outlet exposure and the potential mediator variable neighbourhood social norms (a path). Third, an ordinal logistic regression model with fast food consumption as the outcome variable and fast food outlet exposure and neighbourhood social norms as covariates was fitted to test the independent effects of the mediator (b path) and the exposure (c’ path; direct effect) on the outcome. An attenuation of the direct effect compared to the total effect indicates evidence of mediation. The indirect, or mediated, effect of the exposure on the outcome was calculated as the difference between the total and direct effects (c – c’) [52]. Bootstrapping (1000 replications) was used to calculate percentile-based confidence intervals of the indirect effects [53, 54]. A 95% CI of the indirect effect that does not cross zero indicates evidence of mediation (i.e., a non-zero indirect effect) at a *p* < .05 level. All regression models were adjusted for the confounders age and area-level income.

## Results

### Descriptives

Table 1 shows the descriptive statistics of the sample. The median number of residential fast food outlets was 1.0 (IQR (25th–75th percentile) = 0.0–2.0) and the maximum value was 29.0. On a scale from 1 to 5, respondents had an average score of 2.7 (SD = 0.9) and 3.0 (SD = 0.7) on descriptive and injunctive norms, respectively. In total, 33% of the respondents consumed fast food 1–3 times a month, and 28% consumed fast food at least once a week.

**Table 1:** Descriptive statistics (*N* = 1038)

Age, mean (SD)	45.5 (10.3)
Area level household equivalent income × 1000 euro, mean (SD)	37.3 (7.5)
Fast food outlet exposure (count within 400 m)	
Median (25th–75th percentile)	1.0 (0.0–2.0)
Min-max	0–29
Norm perceptions (scale 1–5), mean (SD)	
Descriptive	2.7 (0.9)
Injunctive	3.0 (0.7)
Fast food consumption, *N* (%)	
Never or less than once/month	413 (39.8)
1–3 time/month	340 (32.8)
At least once a week	285 (27.5)

### Total effect

There was no evidence (*p* = .22) of an overall association between fast food outlet exposure and the odds of fast food consumption (c path, Table 2).

**Table 2:** Mediation analyses results

	Paths								
	a		b		Direct effect c’		Total effect c		Indirect effect c-c’
Mediator	B (95% CI)	*P*	OR (95% CI)	*P*	OR (95% CI)	*P*	OR (95% CI)	*P*	B (95% CI)
Descriptive norm	0.05 (0.03, 0.07)	< .001	1.16 (1.01, 1.33)	.03	1.02 (0.98, 1.06)	.36	1.02 (0.99, 1.07)	.22	0.006 (0.0003, 0.013)
Injunctive norm	0.03 (0.02, 0.04)	< .001	1.44 (1.22, 1.70)	< .001	1.02 (0.98, 1.06)	.46	1.02 (0.99, 1.07)	.22	0.01 (0.004, 0.017)

Note: See Fig. 2 for an overview of the pathways in the mediation model. All analyses were adjusted for age and area-level income. Associations for pathways b, c’, and c are presented on the odds scale (i.e., as odds-ratios), while the indirect effect (c-c’) is presented on the log-odds scale

### Mediation model with descriptive norms as mediator

There was a significant positive association between fast food outlet exposure and descriptive norm perceptions (*p* < .001, a path) (Table 2). Controlling for fast food outlet exposure, perceived descriptive norms were significantly positively associated with the odds of fast food consumption (*p* = .03, b path). Controlling for the mediator descriptive norms, fast food outlet exposure remained non-associated with fast food consumption (*p* = .36, c’ path). There was evidence (at the *p* < .05 level) of an indirect effect of fast food outlet exposure on fast food consumption, via descriptive norms (c-c’).

### Mediation model with injunctive norms as mediator

There was a significant positive association between fast food outlet exposure and injunctive norm perceptions (*p* < .001, a path) (Table 2). Controlling for fast food outlet exposure, perceived injunctive norms were significantly positively associated with the odds of consuming fast food (*p* < .001, b path). Controlling for the mediator injunctive norms, fast food outlet exposure remained non-associated with fast food consumption (*p* = .46, c’ path). There was evidence (at the *p* < .05 level) of an indirect effect of fast food outlet exposure on fast food consumption, via injunctive norms (c-c’).

In sum, the results indicate that both neighbourhood descriptive and injunctive norms may be a mediating pathway in the relation between fast food outlet exposure and consumption.

## Discussion

The present study shows that exposure to fast food outlets in the neighborhood is positively associated with social norm perceptions regarding fast food consumption in the neighbourhood. Moreover, there was evidence that neighbourhood social norms (both descriptive and injunctive) mediated the relationship between fast food outlet exposure and fast food consumption. However, a higher exposure to fast food outlets was not directly associated with higher consumption of fast food. Our findings, although correlational, may suggest that an increased exposure to fast food outlets in the residential neighbourhood may thus shape ‘unhealthier’ norms towards fast food consumption, and these norms may steer actual fast food intake.

This is one of the first studies to demonstrate the pathway by which spatial planning of food outlets may ultimately influence perceptions about food-related code of conduct in a neighbourhood. This association between fast food outlet exposure and neighbourhood norm perceptions (the a path) is intriguing, as it suggests that individuals who have a higher residential availability of fast food outlets, perceive fast food consumption in the neighbourhood as more common and appropriate. It remains unclear however, what specific aspects of fast food outlet exposure may influence norm perceptions. In principal, the exposure measure is purely physical in nature, yet these outlets create opportunities to observe and model others’ consumption behaviours. The general presumption regarding the formation of norms is that social norms are developed through observations of and interactions with others [10, 55], which may be particularly relevant in the context of neighbourhoods, where people live in close proximity with each other [25]. Yet, small-scale experimental studies on diet-related norms showed also that small, physical aspects of the food environment (e.g. empty food wrappers) directly communicate consumption norms [30, 31], and such factors of the residential fast food environment (e.g. empty fast food packaging, meal delivery vehicles) may also steer social norms, yet remains unknown from the current study To our knowledge, the present study is the first to link the structural, neighbourhood-level physical food environment to norm perceptions. Future research may unpack what and how specific neighbourhood-level physical and social aspects influence norm cognitions regarding appropriate consumption.

The positive association found between neighbourhood norms and fast food consumption (the b path) suggests that these perceptions of what is ‘normal’ fast food consumption in the neighbourhood is associated with individual consumption. Humans are part of many different social groups (e.g. family, friends, colleagues) and eating norms may differ between the social groups one belongs to. Although norms of more close relatives may be equally or even more important for one’s eating behaviour [56], our results imply that one’s neighbours are also important for fast food consumption. Effect sizes were small, though on population level these may still be meaningful for eating behaviour. A study on the link between neighbourhood norms about drunkenness and drinking behaviour found that this relationship was significant independent of friend, family, and personal norms [57]. Further research may compare social norms of different reference groups and how they interact in their relation to fast food consumption.

Importantly, it should also be noted that no direct relationship between fast food outlet exposure and consumption was observed. This might be explained from methodological issues. The test of the total effect has relatively low power and therefore it is not uncommon to find an indirect effect even when there is no total or direct effect (See Kenny and Judd for a discussion) [58]. Moreover, there may be unmeasured other mediating pathways, and when varying in sign, they may nullify the overall effect [59]. The lack of evidence for a direct link between fast food outlet exposure and diet/health might also be due to the fact that people may purchase fast food from outside their neighbourhood (e.g. near the workplace, or on the go) [8, 10, 60], thereby undermining the direct influence of fast food outlet that are physically located in the residential neighbourhood on consumption behaviour. However, results of the present study imply that people may eat according to their residential social norms, irrespective of where they purchase their fast food. Accordingly, findings provide preliminary evidence for the proposal of Clary et al. (2017), who suggested that local food outlet exposure may shape preferences and norms that, when progressively internalized, may influence overall food purchasing behaviours [10].

This study has made unique contributions to the literature on the link between fast food environment and diet in two main ways. First, it tested a new conceptual model including perceptions of neighbourhood norms as a mediating pathway between neighbourhood fast food outlet exposure and fast food consumption. Acknowledging that there may be many other mediating pathways, our positive findings contribute to opening the often cited “black box of places” in health and place research [61]. Further research is needed to examine additional pathways (e.g. food preferences) through which the neighbourhood fast food environment may influence consumption. Second, we measured a rather small street-network buffer of 400 m around the home, to study the immediate and visible residential fast food environment. This can be considered a strength for the study purpose. Previous fast food access studies that employed buffer metrics commonly used larger buffers ranging from 800 m to 3 km around the home address, whereas smaller buffers of 400 m have predominantly been used around schools [8, 9, 13, 62, 63]. Yet, a small buffer of 400 m around the residential address was considered relevant for the formation of an individual’s immediate neighbourhood norm perceptions, because a direct and daily/frequent exposure to the residential food environment may enhance internalisation of norms in the neighbourhood (see also [10]). It remains to be tested if results are generalizable to other countries, as street networks and types of fast food outlets differ over countries.

This study has several other noteworthy methodological strengths. First, we included a national representative sample with respect to sex, age, education level, and province. Second, by distributing the survey in two waves with a four weeks interval we aimed to prevent that responses to the first set of items (including psychological measures, e.g. neighbourhood social norms) would influence responses to the second set (including fast food consumption). Third, the use of DAGs allowed us to carefully identify necessary adjustment for confounders while avoiding overadjustment, which may in itself introduce bias [43].

This study also has some limitations. First, as this study has a correlational design, no causal conclusions can be drawn. The assumed direction of the relationship between fast food outlet exposure, neighbourhood norms and fast food consumption may also be reversed. For example, the act of making inferences about the frequency of others’ fast food consumption may be biased by own fast food consumption (confirm the false consensus effect). Moreover, fast food outlet exposure might be biased by neighbourhood self-selection. Therefore, natural experiments examining changes in the residential fast food environment (e.g. [64]) are needed to further explore the mediating role of changes in neighbourhood norms in the impact on fast food consumption. Nevertheless, it was deemed implausible that people determine their home location based on residential fast food availability. Second, the dates of data collection of the fast food outlet exposure measure (end of 2017) and the norms and consumption measures (early 2019) did not align exactly. However, it is unlikely that the minor changes in the availability of fast food outlets influenced the results drastically. Third, due to privacy reasons we could not obtain the exact address of the respondents. However, a postcode area in the Netherlands represents on average 25 houses and would closely represent exposure at the precise address point. Fourth, the social norm items were only framed in a positive direction. Disagreement about items measuring the appropriateness of fast food consumption could imply that respondents perceived an ‘opposing’ norm or that no norm was perceived whatsoever. Using a negative framing would be interesting to verify our findings: when *less* exposed to fast food outlets, do people find fast food consumption more *un*common and *in*appropriate? Fifth, the fast food consumption measure relied on self-report and recall which must be taken into account in the interpretation of the findings. Yet, a FFQ has been shown to be a valid and practical tool to provide a reasonable accurate ranking of low to high food intake [65, 66].

## Conclusions

The present study provided the first evidence for the mediating role of neighbourhood fast food norms in the much studied association between neighbourhood fast food outlet exposure and fast food consumption. Acknowledging the correlational design, results imply that a higher exposure to fast food outlets in the residential neighbourhood may form ‘unhealthier’ norms regarding fast food consumption, and these norms may guide fast food consumption. The food environment is rapidly changing: apart from fast food outlets, the number of full-service restaurants, coffee shops, and convenience stores increased over time in residential as well as workplace and commuting environments [67, 68]. Hence, we invite future research to test the mediating role of social norms in different food contexts and to disentangle how these may shift our norms regarding common and appropriate consumption. Such insights would support policymakers in urban planning to develop healthier neighbourhoods and ultimately stimulate healthier consumption.

## Supplementary information

**Additional file 1.** DAG representing assumed causal pathways between fast food outlets, fast food consumption and covariates. Created with Dagitty [69].

## Data Availability

The datasets generated and analysed during the current study are not publicly available due to privacy reasons as stated in the informed consent but are available from the corresponding author on reasonable request.
