# Supplementary figures and images for: Neighbourhood fast food exposure and consumption: the mediating role of neighbourhood social norms
Source: Int J Behav Nutr Phys Act. 2020 May 13;17:61. doi: 10.1186/s12966-020-00969-w (PMC7218623; doi:10.1186/s12966-020-00969-w)

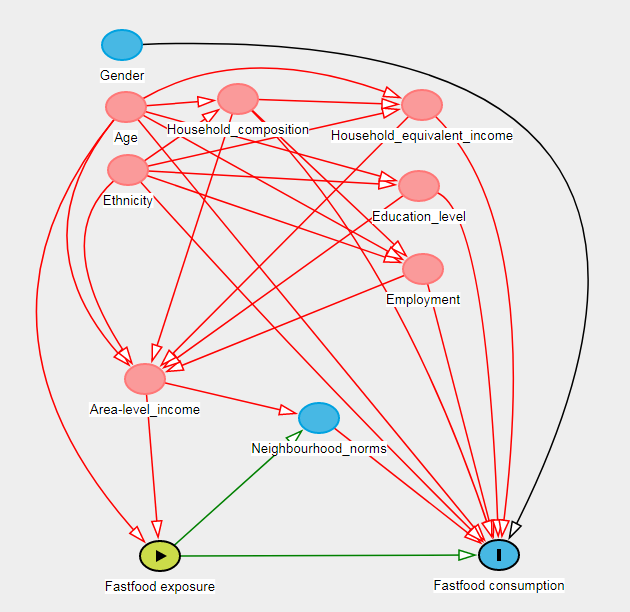

Supplement: Supplementary file 1 — Additional file 1. DAG representing assumed causal pathways between fast food outlets, fast food consumption and covariates. Created with Dagitty [69]. [file 12966_2020_969_MOESM1_ESM.png]
